# FDG-PET/CT for differentiating between aseptic and septic delayed union in the lower extremity

**DOI:** 10.1007/s00402-017-2806-8

**Published:** 2017-09-27

**Authors:** Kirsten E. van Vliet, Vincent M. de Jong, M. Frank Termaat, Tim Schepers, Berthe L. F. van Eck-Smit, J. Carel Goslings, Niels W. L. Schep

**Affiliations:** 1 Trauma Unit, Department Surgery, AMC, Meibergdreef 9, 1105 AZ Amsterdam, The Netherlands; 2 Trauma Unit, Department of Surgery, LUMC, Leiden, The Netherlands; 3 Department of Nuclear Medicine, AMC, Amsterdam, The Netherlands; 4 Trauma Unit, Department of Surgery, Maasstad ziekenhuis, Rotterdam, The Netherlands

**Keywords:** FDG-PET, Delayed fracture healing, Bone infection, Osteomyelitis, Lower extremity

## Abstract

**Background:**

^18^F-fluorodeoxyglucose (^18^F-FDG) positron emission tomography (PET)/computed tomography (CT) has proven to have a high diagnostic accuracy for the detection of bone infections. In patients with delayed union it may be clinically important to differentiate between aseptic and septic delayed union. The aim of this study was to evaluate the efficacy and to assess the optimal diagnostic accuracy of FDG-PET/CT in differentiating between aseptic and septic delayed union in the lower extremity.

**Methods:**

This is a retrospective study of consecutive patients who underwent FDG-PET/CT scanning for suspicion of septic delayed union of the lower extremity. Diagnosis of aseptic delayed union or septic delayed union was made based on surgical deep cultures following PET/CT scanning and information on clinical follow-up. FDG-uptake values were measured at the fractured site by use of the maximum standardized uptake value (SUV_max_). Sensitivity, specificity and diagnostic accuracy of FDG-PET/CT were calculated at various SUV_max_ cut-off points.

**Results:**

A total of 30 patients were included; 13 patients with aseptic delayed unions and 17 patients with septic delayed unions. Mean SUV_max_ in aseptic delayed union patients was 3.23 (SD ± 1.21). Mean SUV_max_ in septic delayed union patients was 4.77 (SD ± 1.87). A cut-off SUV_max_ set at 4.0 showed sensitivity, specificity and diagnostic accuracy of FDG-PET/CT were 65, 77 and 70% to differentiate between aseptic and septic delayed union, respectively.

**Conclusion:**

Using a semi-quantitative measure (SUV_max_) for interpretation of FDG-PET/CT imaging seems to be a promising tool for the discrimination between aseptic and septic delayed union.

## Background

Osteomyelitis is an inflammatory process of bone caused by microorganisms. Microorganisms such as Staphylococcus aureus (30%) and coagulase-negative staphylococci (22%) are the most common pathogens found in patients with osteomyelitis; however, poly-microbial cultures are found in 29% of the cases [1, 2].

Diagnosis of osteomyelitis remains difficult due to the absence of clear clinical, radiological and laboratory findings [3]. Laboratory parameters, such as CRP and leukocyte count, lack the sensitivity and specificity to diagnose osteomyelitis [4]. Standard follow-up with conventional radiography has a low sensitivity varying from 43 to 75% and specificity from 75 to 83% to detect osteomyelitis. Positive findings on radiographs are reliable, but indicate a late stage of osteomyelitis since abnormalities are not detectable until 50–75% of the bone matrix is destroyed [5, 6]. This lack of diagnostic accuracy requires an alternative diagnostic approach in trauma patients.

A previously published meta-analysis showed that ^18^F-fluorodeoxyglucose (^18^F-FDG) positron emission tomography (PET)/computed tomography (CT) scan showed the highest diagnostic accuracy (sensitivity 96% and specificity 91%) for suspected osteomyelitis, compared with other imaging modalities, such as bone scintigraphy, leucocyte scintigraphy and MRI [7]. Uptake of FDG is a reflection of cellular glucose metabolism and is semi-quantitatively expressed as the standardized uptake value (SUV). Physiological uptake is present in all tissues of the body and thus in bone as well. Increased FDG-uptake occurs in areas with increased glucose consumption due to increased metabolic activity of the tissue involved or due to the invasion of inflammatory cells [8–10]. Hybrid PET and CT combines detection of metabolic changes with morphologic information and may therefore be helpful in the assessment of post-traumatic osteomyelitis.

However, the clinical problem is to differentiate between delayed union with and without the presence of osteomyelitis, as increased metabolism and morphologic changes probably will be present in both conditions [11, 12]. Considering the fact that osteomyelitis is almost always accompanied by delayed fracture healing, the term ‘septic delayed union’ can be better used for the condition of bone described in this study. We hypothesize that in case of septic delayed union metabolism will be increased to a higher degree than in the case of aseptic delayed union. Therefore the metabolic activity on FDG-PET/CT, the standardized uptake value (SUV_max_), was assessed in a cohort of consecutive patients with suspicion of septic delayed union.

The aim of this study was to evaluate the efficacy and to assess the optimal diagnostic accuracy of FDG-PET/CT in differentiating between aseptic and septic delayed union in the lower extremity.

## Methods

### Patient population

A retrospective study was performed in a cohort of consecutive patients undergoing FDG-PET/CT scanning for the suspicion of septic delayed union. All patients were selected from an electronic database at our academic Level-1 trauma center from the period March 2010 to October 2014. Patients were excluded if they underwent surgery within 3 months to PET/CT, because it is known that FDG-levels will normalize within approximately 3 months after surgery [9, 13].

## Methods

Suspicion of septic delayed union was based on clinical signs, mostly unexplained pain. The diagnosis aseptic or septic delayed union was made based on surgical deep cultures after PET/CT scanning. Aseptic delayed union was defined as two negative deep cultures or a negative clinical follow-up of 1 year. Septic delayed union was defined as at least one positive deep culture out of two samples or a positive clinical follow-up of 1 year. The presence of a sinus tract or pus was diagnosed as septic delayed union (Table 1).

**Table 1:** Patients’ characteristics

Patient no./sex/age (years)	Fracture site	Culture	Final diagnosis
1/M/29	Tibia	Negative	Aseptic delayed union
2/M/47	Tibia	Positive	Septic delayed union
3/M/63	Tibia	No culture	Aseptic delayed union
4/F/51	Tibia	Positive	Septic delayed union
5/F/74	Calcaneus	Positive	Septic delayed union
6/M/51	Calcaneus	Positive	Septic delayed union
7/M/25	Femur	Positive	Septic delayed union
8/F/34	Tibia	Negative	Aseptic delayed union
9/F/23	Femur	No culture	Aseptic delayed union
10/M/18	Tibia	Positive	Septic delayed union
11/F/30	Calcaneus	Negative	Aseptic delayed union
12/F/73	Tibia	No culture	Aseptic delayed union
13/M/47	Calcaneus	Negative	Aseptic delayed union
14/M/31	Tibia	No culture	Septic delayed union
15/M/39	Tibia	No culture	Aseptic delayed union
16/M/42	Tibia	Negative	Aseptic delayed union
17/F/64	Calcaneus	Positive	Septic delayed union
18/M/33	Tibia	Negative	Aseptic delayed union
19/M/57	Tibia	Positive	Septic delayed union
20/M/47	Tibia	No culture	Septic delayed union
21/M/60	Tibia	Positive	Septic delayed union
22/M/68	Tibia	Positive	Septic delayed union
23/M/70	Tibia	No culture	Septic delayed union
24/M/49	Tibia	Negative	Aseptic delayed union
25/F/56	Calcaneus	Positive	Septic delayed union
26/M/41	Tibia	Positive	Septic delayed union
27/F/39	Tibia	Negative	Aseptic delayed union
28/M/25	Femur	Positive	Septic delayed union
29/M39	Femur	Positive	Septic delayed union
30/M/55	Tibia	Negative	Aseptic delayed union

For the assessment of FDG-uptake in the bone we used the maximum standardized uptake value (SUV_max_), which is a semi-quantitative measure for FDG-uptake. The region of interest (ROI) was determined by identifying the affected region on CT. SUV_max_ in this region was determined on the transaxial PET images using Hermes Hybrid PDR v 1.4B (HERMES Medical Solutions AB, Stockholm, Sweden). FDG-uptake values in normal contralateral bone were measured as well. The ROI was drawn on transaxial CT images around the cortex and superimposed on the corresponding PET image were SUV_max_ was calculated. The normal contralateral bone was used as a reference. In patients with bilateral fractures, comparisons with the contralateral site were omitted.

For the efficacy of FDG-PET/CT to differentiate between aseptic and septic delayed union, we calculated the sensitivity, specificity and diagnostic accuracy at various SUV_max_ cut-off points.

### PET/CT scanning

Each patient underwent ^18^F-FDG-PET-CT on a Philips Gemini-16 TOF PET-CT scanner (Philips Healthcare, Eindhoven, The Netherlands). Based on BMI a dose of 180-400 MBq18F-FDG was administered intravenously. Before administration of the radio-tracer patients fasted for at least 6 h and serum glucose level was checked to be < 10 mmol/l. Patients had bed rest from 15 min before till 30 min after administration of FDG in a room of at least 23 °C. FDG-PET-CT imaging started 60 min ± 10 min post injection.

Images were acquired with a low-dose PET-CT protocol (CT: 120 kV, 60mAs, 16 × 1.5 collimation, 0.813 pitch; PET: 2 min/bed position) and reconstructed with standard Philips time-of-flight reconstruction software (3D LOR OSEM).

Transverse, coronal and sagittal image reconstructions were performed with and without attenuation correction.

### Statistical analysis

The statistical analysis was performed using SPSS, v.20 (SPSS for windows, version 20; SPSS^®^Inc., Chicago, Illinois, USA). Normality of continuous data was tested with the Shapiro–Wilk and Kolmogorov–Smirnov test and by inspecting the frequency distributions (histograms). Homogeneity of variance was tested using the Levene’s test.

Descriptive analysis was performed to assess baseline characteristics, medians and percentiles (non-parametric data) and means and standard deviations (parametric data) were calculated. Differences were assessed using the Student’s *T* test (parametric data) or the Mann–Whitney *U* test (non-parametric data). Categorical data were compared using the Chi-square test. *P* value < 0.05 was taken as the threshold of statistical significance.

Sensitivity, specificity and diagnostic accuracy to differentiate between septic delayed union and aseptic delayed union were calculated for different SUV_max_ uptake values. The accuracy of PET/CT for diagnosing septic delayed union at various SUV_max_ cut-off points were plotted in a receiver operating characteristic (ROC) curve, in which the test characteristics of sensitivity and specificity were calculated to discriminate between aseptic and septic delayed union.

## Results

**Fig. 1:**
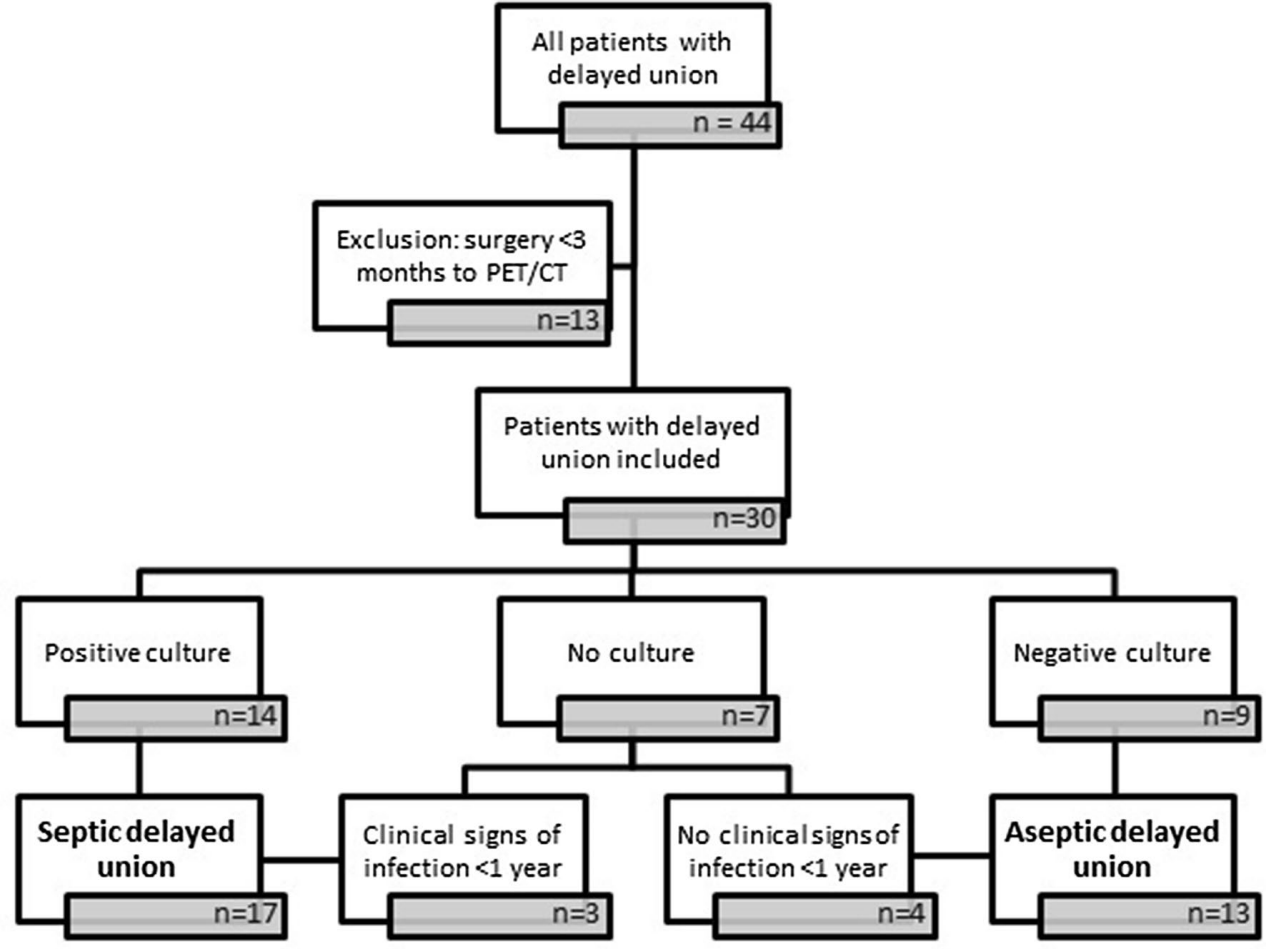
Flowchart of methods: all patients were divided into aseptic or septic delayed union based on surgical deep cultures or clinical follow-up

Thirty patients were included, 21 men and 9 women with a mean age of 46 years (range 18–74); 4 patients had a femur fracture, 20 patients a tibial fracture, and 6 patients a calcaneal fracture. Final diagnosis demonstrated a total of 13 aseptic delayed unions and 17 septic delayed unions (Fig. 1; Table 2). Two patients had bilateral fractures. Of 28 patients, contralateral normal bones were measured as well.

**Table 2:** Summary of patients’ characteristics and SUVmax

Patients’ characteristics and SUVmax			
No. of patients	30		
Sex (M/F)	21/9		
Mean age (years)	46 (range 18–74)		
	Aseptic delayed union	Septic delayed union	
No. of fractures	13	17	
Location of fracture:			
Femur	1	3	
Tibia	10	10	
Calcaneus	2	4	
SUVmax:			
Mean	3.23	4.77	*P* value
SD	1.21	1.87	0.016

Fourteen patients in the septic delayed union group had positive perioperative culture. Bacteria found in the bacteriological cultures were mostly anaerobic bacteria, such as Enterococcus, Escherichia, Fusobacterium and Clostridium and Staphylococcus bacteria or a combination of these bacteria.

### Standardized uptake values

Aseptic delayed union patients had a mean of SUV_max_ 3.23 (SD ± 1.21). Septic delayed union patients had a mean of SUV_max_ 4.77 (SD ± 1.87). SUV_max_ in aseptic delayed union was significantly lower compared to septic delayed union (*P* value 0.016). Contralateral normal unaffected bones had a mean of SUV_max_ 0.76 (SD ± 0.26).

**Fig. 2:**
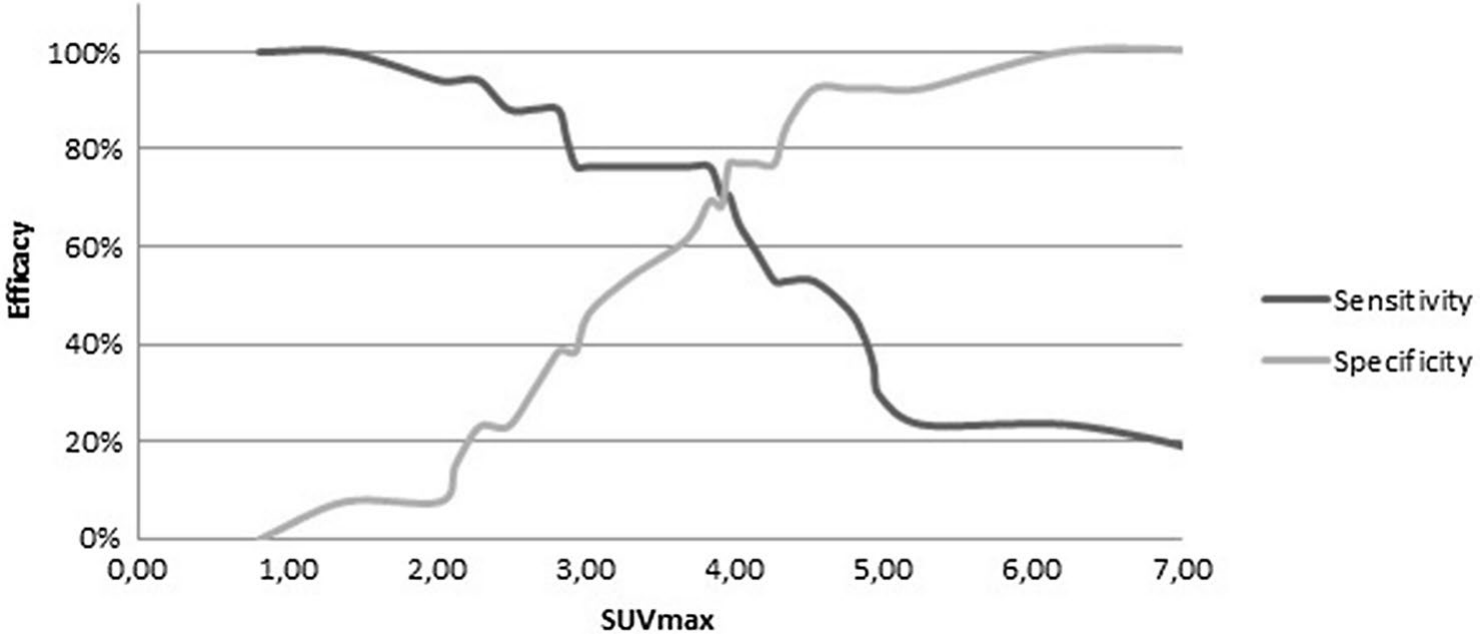
The efficacy of FDG-PET/CT to discriminate between aseptic and septic delayed union at various SUV_max_ cut-off points. Lines cross approximately at SUV_max_ 4.0 which corresponds with a sensitivity of 65% and specificity of 77%

The efficacy of FDG-PET/CT to discriminate between aseptic and septic delayed union at various SUV_max_ cut-off points were plotted into a ROC curve (Fig. 2). Sensitivity, specificity and diagnostic accuracy of FDG-PET/CT were 0.65, 0.77 and 70.0, respectively and an AUC of 0.747, with a cut-off SUV_max_ set at 4.0 (Table 3).

**Table 3:** Efficacy of FDG-PET/CT to differentiate between aseptic and septic delayed union at SUV_max_ cut-off points

SUV_max_ cut-off	Septic delayed union (*n* = 17)		Aseptic delayed union (*n* = 13)				
	True positive	True negative	False positive	False negative	Sensitivity	Specificity	Diagnostic accuracy
> 3.0	13	5	8	4	0.76	0.38	0.60
> 4.0	11	10	3	6	0.65	0.77	0.70
> 5.0	5	12	1	12	0.29	0.92	0.57

## Discussion

This retrospective study showed that aseptic delayed union is characterized by a lower metabolic activity than septic delayed union. Integrating this metabolic activity, expressed as SUV_max_ in the interpretation of the images helps in the differentiation of both conditions. In this study of 30 delayed union fractures, 13 aseptic delayed unions had a mean SUV_max_ of 3.23 and 17 septic delayed unions had a mean SUV_max_ of 4.77, which is a significant difference (*P* value 0.016). Normal contralateral bones had a mean SUV_max_ of 0.76.

For the use of the FDG-PET/CT scan as a diagnostic tool, it is essential to weight the importance of discriminating and excluding septic delayed union from aseptic delayed union in trauma patients. Higher sensitivity may lead to unnecessary surgery, while higher specificity could cause undertreatment of septic delayed union. A cut-off SUV_max_ set at 4.0 indicates an approximately equal and acceptable sensitivity and specificity of 65 and 77%, respectively. SUV_max_ set at 3.0 indicates a good sensitivity of 76%, but simultaneously gains at the expanse of the specificity reducing to 38%. Higher SUV_max_ of 5.0 has an excellent specificity of 92%, but has a very poor sensitivity of 29% in contrast. Therefore, in our opinion a SUV_max_ set at 4.0 is the best cut-off point for the use of this diagnostic tool.

To understand the process of FDG-uptake in case of abnormal bone healing, one should take into account the dynamics of normal bones and uncomplicated fracture healing expressed by FDG-uptake. In our first study, we therefore determined the FDG uptake in normal bones. We concluded that normal FDG uptake of the long bones of the lower extremity have a SUV_mean_ < 0.5 and a SUV_max_ < 0.8 [14]. Two studies have described the patterns of physiological FDG-uptake in bones during uncomplicated fracture healing. Zhuang et al. demonstrated in 37 patients that FDG-uptake may be normal within 12 weeks following fracture [15]. Shon et al. reported a study of four patients with uncomplicated fracture healing that FDG-uptake normalized within 8 weeks [16]. These two studies confirm that FDG-uptake of physiological bone healing will normalize in time based on visual assessment of the PET image. Unfortunately, no studies have been published defining the FDG-uptake pattern, let alone SUV measures, in the process of complicated fracture healing. In the setting of complicated fracture healing such as aseptic delayed fracture healing and septic delayed union, reasons for increased metabolism results into poor differentiation between the origin of increased FDG-uptake. In our study we included patients with delayed fracture healing to differentiate septic delayed union from aseptic delayed union.

Some discrepancies were found in the rates of diagnostic accuracy parameters between this study and previous studies. Schiesser et al. described a sensitivity, specificity and diagnostic accuracy of 100, 87.5 and 95% respectively for detection of osteomyelitis. This study included soft tissue infections as well, which could explain the higher diagnostic accuracy. Noteworthy, one of the false-positive cases described in this study turned out to be a delayed union [17]. Winter et al. found a sensitivity, specificity and diagnostic accuracy of 100, 86 and 93% respectively for detection of osteomyelitis in the peripheral skeleton. Two of four false positive findings were related to recent surgery within the previous 6 months [13].

Hartmann et al. used visual assessment and found a sensitivity, specificity and diagnostic accuracy of 100, 85 and 91% respectively for detection of osteomyelitis in the peripheral skeleton. FDG-uptake was expressed into intensity of greyscale and was graded into a five-point scale [18]. Wenter et al. also used visual assessment in addition to SUV measurements and found a sensitivity, specificity and diagnostic accuracy of 85, 86 and 86% respectively [19]. In our opinion, the visual scoring of FDG-uptake is less objective than the use of SUVs and may be more sensitive to inter- and even intraobserver differences. Therefore we call for an unbiased predictive SUV cut-off point to differentiate between normal bone uptake, uncomplicated fracture healing, aseptic delayed union and septic delayed union.

Guhlmann et al. did use SUV measurements for FDG-uptake for diagnosing osteomyelitis. This study found a very high sensitivity, specificity and diagnostic accuracy; 100, 92 and 97%. The high diagnostic accuracy found in this study can be explained by the long follow-up of patients in whom ongoing bone healing can be excluded and the risk for more fulminant osteomyelitis increases. Mean SUV found in peripheral fractures with osteomyelitis was 3.6 (SD 2.0), which approaches the SUV found in our study; SUV_max_ 4.77 [20]. However, it is not consistent to compare these results with our results, because it is unclear if they used SUV_mean_ of SUV_max_ to calculate the FDG-uptake.

Overall it can be stated that in previous studies a higher diagnostic accuracy of FDG-PET/CT for detecting osteomyelitis has been found compared to our diagnostic accuracy for detecting septic delayed union. This can be explained by differences in patient population and the different methods to quantify FDG-uptake.

Our data demonstrate that FDG-PET/CT is able to differentiate between aseptic and septic delayed union in the lower extremities in trauma patients by using the cut-off SUV_max_ set at 4.0. These findings broaden the domain in which FDG-PET/CT can provide additional information for guiding surgical therapy into an earlier and clinical relevant detection period. Early diagnosis has an obvious advantage for guiding surgical treatment which will lead to reduced morbidity compared to an unrecognized septic delayed union.

A larger prospective cohort study is needed to establish the distribution pattern of FDG-uptake during fracture healing in order to develop a diagnostic window for FDG-PET/CT in septic delayed union after trauma and osteosynthesis.

## Limitations and strengths

Drawback of this study is its retrospective design with all its known sources of bias and the small number of patients included due to low prevalence of septic delayed unions. Moreover, we have just one assessment of FDG-uptake in time and therefor nothing can be concluded about the dynamics of the process.

The strength of our study is that there has been limited research about the FDG-uptake in fractures of the lower extremity, while this information could be important in early detection of septic delayed union after post-traumatic treatment to prevent the bone from (irreversible) damage. Another important aspect of the use of an objective measure such as SUV is the reduction of interobserver discrepancies in the assessment of septic delayed union.

## Conclusion

Using a semi-quantitative measure (SUV_max_) for interpretation of FDG-PET/CT imaging seems to be a promising tool for the discrimination between aseptic and septic delayed union. To use the FDG-PET/CT as a diagnostic tool, larger prospective studies with multiple measurements in the course of fracture healing should be executed.
